# Population genetic diversity in zebrafish lines

**DOI:** 10.1007/s00335-018-9735-x

**Published:** 2018-01-24

**Authors:** Michele Balik-Meisner, Lisa Truong, Elizabeth H. Scholl, Robert L. Tanguay, David M. Reif

**Affiliations:** 1 Bioinformatics Research Center, Center for Human Health and the Environment, Department of Biological Sciences, North Carolina State University, Ricks Hall 344, 1 Lampe Drive, Box 7566, Raleigh, NC 27695 USA; 2 Sinnhuber Aquatic Research Laboratory, Department of Environmental and Molecular Toxicology, Oregon State University, Corvallis, OR 97331 USA

## Abstract

Toxicological and pharmacological researchers have seized upon the many benefits of zebrafish, including the short generation time, well-characterized development, and early maturation as clear embryos. A major difference from many model organisms is that standard husbandry practices in zebrafish are designed to maintain population diversity. While this diversity is attractive for translational applications in human and ecological health, it raises critical questions on how interindividual genetic variation might contribute to chemical exposure or disease susceptibility differences. Findings from pooled samples of zebrafish support this supposition of diversity yet cannot directly measure allele frequencies for reference versus alternate alleles. Using the Tanguay lab Tropical 5D zebrafish line (T5D), we performed whole genome sequencing on a large group (*n* = 276) of individual zebrafish embryos. Paired-end reads were collected on an Illumina 3000HT, then aligned to the most recent zebrafish reference genome (GRCz10). These data were used to compare observed population genetic variation across species (humans, mice, zebrafish), then across lines within zebrafish. We found more single nucleotide polymorphisms (SNPs) in T5D than have been reported in SNP databases for any of the WIK, TU, TL, or AB lines. We theorize that some subset of the novel SNPs may be shared with other zebrafish lines but have not been identified in other studies due to the limitations of capturing population diversity in pooled sequencing strategies. We establish T5D as a model that is representative of diversity levels within laboratory zebrafish lines and demonstrate that experimental design and analysis can exert major effects when characterizing genetic diversity in heterogeneous populations.

## Introduction

Use of the zebrafish (*Danio rerio*) as a model organism has gained momentum in vertebrate genomics (Lieschke and Currie 2007). As a vertebrate with one of the largest sets of protein-coding genes, consisting of orthologues for over 70% of human genes, they have been adapted as exposure and human disease models (Howe et al. 2013). There are many benefits to using zebrafish in developmental studies, including early maturation as clear embryos that are amenable to easily observable morphological endpoints, short generation time, and well-characterized development that is conserved across species during the phylotypic period (Kimmel et al. 1995; Irie and Kuratani 2011). These advantages have led to an upward trend in high-throughput zebrafish chemical screens, especially toward screens of many chemicals using large quantities of fish (Usenko et al. 2007; Bai et al. 2009; Truong et al. 2014; Asharani et al. 2015). Thus, this model could be used for large-scale studies of chemical bioactivity that include genetic information on response mechanisms during development of exposed individuals (Baer et al. 2014) or even across multiple generations (Kovács et al. 2015; Knecht et al. 2017).

Model organisms have long been utilized to study genetic determinants underlying human disease susceptibility, because experiments can exert necessary controls over factors such as diet, lifestyle, and environment that would be impossible in a human setting. The mouse has been extensively used to mechanistically model human disease, but until the inception of a major recombinant inbred line (RIL) panel, the lack of variability within any single inbred strain did not sufficiently model human genetic variability (Churchill et al. 2004). The RIL strategy had been implemented multiple times in mice, but their utility was insufficiently broad due to limited genetic diversity in lines stemming from two inbred strains. In order to create a RIL panel representing the genetic diversity among a more general populace of mice, the collaborative cross (CC) (Chesler et al. 2008) was implemented to randomly mix the genomes of eight founder strains to create hundreds of isogenic RILs (Churchill et al. 2004). The eight founder strains included five classical inbred strains and three wild-derived strains that jointly capture 90% of the known allelic diversity in the mouse genome (Roberts et al. 2007). A RIL strategy aiming to capture diversity has also been used in fruit flies (*Drosophila melanogaster*) (Mackay et al. 2012). For these populations, each isogenic line has been sequenced. Individuals within one line are homogeneous, but comparisons of traits or susceptibility between lines have aided in identifying genetic associations (Cirelli et al. 2008; Unckless et al. 2015; Ivanov et al. 2015).

Nonetheless, isogenic models of any species fail to model the influence of genetic diversity on toxicity responses, a critical factor in human responses to toxicants. As noted by French et al., “Inadvertent selection of a strain with an idiosyncratic response could result in significant bias and compromise the reliability of safe exposure estimates” (2015). In order to use the CC mice in an infrastructure more similar to naturally occurring populations with heterozygosity, an outbred population was created. The diversity outbred (DO) population was derived from 144 CC lines at various stages (4–12 generations) of inbreeding, allowing recombination events in the early generations to promote recombination and genetic diversity amongst the DO mice (Svenson et al. 2012). Approximately 45 M single nucleotide polymorphisms (SNPs) segregate in the CC and DO populations, four times more than in any singular laboratory mouse strain (Yang et al. 2011). Each DO individual is unique and cannot be precisely replicated, but haplotypes can be reconstructed based on the determination of recombination events using knowledge of the CC founder strain homozygous genotypes, and CC mice can be used to test hypotheses generated through use of DO mice (Churchill et al. 2012). When employed appropriately, these resources can provide insight on a number of variants that should be more in line with that found in a wild-type (WT) population.

In zebrafish, inbreeding adversely affects fecundity and survival (Mrakovcic and Haley 1979), so endeavors to create isogenic lines have not been fruitful. Zebrafish populations differ from many model organisms in that the standard husbandry practices are often designed to maintain diversity (Nasiadka and Clark 2012). Thus, like human populations, most laboratory zebrafish populations contain an unknown level of genetic diversity (Brown et al. 2012). Comparisons between named strains and inter-lab populations of zebrafish have shown variability in several phenotypes, providing the rationale that constitutive genetic variation may contribute to the variability in exposure response (Lange et al. 2013). Despite the small samples (1–2 individual fish or relatively small, pooled samples) used in studies aiming to characterize genetic diversity, results have shown between 5 and 15 million SNPs segregating in a zebrafish population, with roughly half of the variants showing evidence of population-specificity (Obholzer et al. 2012; Patowary et al. 2013; LaFave et al. 2014; Butler et al. 2015). It has been estimated that zebrafish populations have a larger abundance of SNPs per kb of unique sequence than ethnically defined human populations (Butler et al. 2015).

Here, we characterize salient features of population genetic architecture of the Tropical 5D (T5D) line as a representative laboratory population of zebrafish. The T5D line is an “outbred” population of heretofore unknown genetic heterogeneity that has been used to screen thousands of chemicals for adverse biological responses (Truong et al. 2014; Reif et al. 2016). We obtained whole genome sequences of 276 individuals from the T5D population, aligned reads to the GRCz10 reference genome, called SNPs and indels, and created a T5D-specific reference genome. This was performed with the aims of characterizing genomic variability in the outbred, T5D wild-type zebrafish population, discovering the type of variation (common SNPs versus rare variants, etc.) observable in the population, and establishing the validity of the T5D population as a heterogeneous model. We then empirically compared genomic characteristics of our zebrafish population with murine and human reference populations, as well as across other zebrafish lines. Finally, we explored whether the higher apparent diversity observed in our T5D line could be due to experimental design factors that tend to underestimate diversity in other published lines.

## Materials and methods

### Datasets and variant consequence predictions for interspecies comparisons

**Fig. 1:**
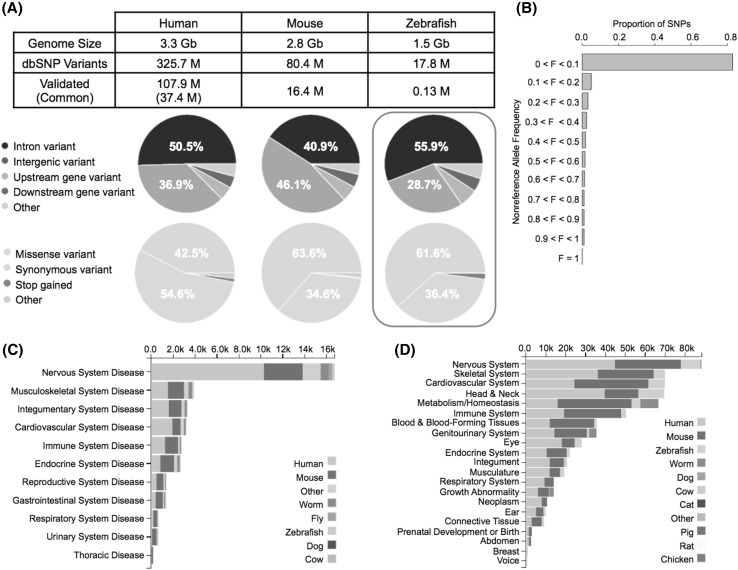
Known variants. **a** Genome size, known variant count in dbSNP, variant effect, and consequences of transcript variants. The red box contains the variant effects for the 20.1 M SNPs found in T5D. (All other zebrafish data refer to the reference genome and publically available data). **b** Allele frequency spectrum for common human variants. **c** Number of models per disease category stacked by organism (from https://monarchinitiative.org). **d** Number of phenotype-gene associations per species (from monarchinitiative.org)

Short genetic variation datasets for human, mouse, and zebrafish from NCBI’s dbSNP were downloaded from ftp://ftp.ncbi.nih.gov/snp/organisms/. The effect of the variants on genes and transcripts and consequences on protein sequence were annotated for each species using Ensembl variant effect predictor (VEP) (McLaren et al. 2016) (Fig. 1). Genome size and statistics on variant counts and distributions were compared across species.

### Developmental screening system and experimental population

The T5D founders of our experimental population were originally imported into the Tanguay lab at Oregon State University from a breeding facility containing thousands of zebrafish in 2007 to generate a Pseudoloma neurophilia (Microsporidia) free line (Stanley et al. 2009). The T5D zebrafish are housed at Sinnhuber Aquatic Research Laboratory (SARL) at Oregon State University and maintained in accordance with their Institutional Animal Care and Use Committee protocols. Fish are raised in a recirculating water system with a temperature of 28 ± 1 °C and a 14-h light: 10-h dark photoperiod. All generations are propagated with equal proportions of offspring contributed from a minimum of 25 small group crosses, each group containing three males and up to three females.

### Genotyping by sequencing

The sequencing data are described in detail in (Balik-Meisner et al., *submitted*). In brief, genomic DNA was extracted (Zymo Quick-DNA 96-Kit Cat # D3011) from 276 individual larvae exposed to 0.6 µM Abamectin at 120-h post fertilization. The authors note that Abamectin is non-genotoxic (Oliveira et al. 2016), so exposure would not have altered constitutive DNA sequence. The extraction protocol was followed according to the manufacturer and DNA was eluted in water. All library preparation and sequencing were performed at Oregon State University’s Center for Genome Research and Biocomputing (http://cgrb.oregonstate.edu/core). For these samples, 350 ng of DNA was used in the library preparation. Prior to library prep, the quality and quantity were verified using a fluorometric plate reader and Bioanalyzer. Samples were sheared to ~ 320 bp, and 100 ng was used in the WaferGen robotic DNA library prep. After the library prep, each sample was quantified to verify similar input for sequencing. The samples were sequenced on an Illumina HiSeq 3000 with 12 samples per lane (~ 5× coverage) and 150 bp paired-end sequencing.

### Alignment

FastQC output indicated that reads were 151 bps in length. GC content for each sample was ~ 37%, which is consistent with the zebrafish genome (Han and Zhao 2008). For each sample (DNA from an individual zebrafish), reads were aligned to the Genome Reference Consortium GRCz10 (Howe et al. 2013) reference genome with Bowtie 2 (Langmead and Salzberg 2012) using standard settings. The overall alignment rate was ~ 89% for each sample. Potential PCR duplicates were then removed using Samtools rmdup (Li et al. 2009).

### Variant calling and filtering

Variant calls were generated for each individual at every variant site. A variant call was made at any site (across the entire genome, including all chromosomes and mitochondrial DNA, excluding non-chromosomal material or scaffolds not aligned within a chromosome), where there was sufficient evidence (based on reads, quality scores, etc.) of a non-reference base for at least one individual. GATK (Mckenna et al. 2010) HaplotypeCaller was used to call genotypes on all samples simultaneously (joint genotyping). This leverages data across samples to assign genotypes for individuals with low coverage at certain bases using a Bayesian likelihood model for genotyping. Reads with a mapping quality below 20 were not included, and a minimum phred-scaled confidence threshold of 10 was required. Genotypes are reported for every individual at every variant site for which they had any remaining reads.

Before base quality control/filtration, there were 36,532,474 SNPs and 7,262,723 indel variants with an average of 4.2× coverage per site. The GATK Variant Filtration tool was used to implement the GATK best practices (Depristo et al. 2011) hard filtering recommendations for SNPs and indels (filter SNPs with quality by depth (QD) < 2, phred-scaled Fisher’s exact test p-value (FS) > 60, root mean square mapping quality (MQ) < 35, mapping quality Mann–Whitney Rank-Sum < − 12.5, or read position Mann–Whitney Rank-Sum < − 8, strand odds ratio (SOR) > 3; filter indels with QD < 2, FS > 100, read position Mann–Whitney Rank-Sum < − 20, SOR > 10). The adjustment of the MQ threshold from GATK’s recommendation of 40 to 35 accounted for the difference in quality score reporting between the aligner suggested by GATK (BWA) and Bowtie 2. BWA outputs a larger range of mapping quality scores, averaging 60 for high confidence reads, whereas the maximum quality score for Bowtie 2 is 42, indicating a perfectly aligned read. After applying the filtering cutoffs, 20,385,817 SNPs and 6,304,066 indels remained.

### Variant consequence predictions for interspecies comparisons to T5D zebrafish

VEP (McLaren et al. 2016) was also run on the set of T5D variants to determine their predicted effects and consequences. These results were compared to the other species (human and mouse).

### Variant set preparation for zebrafish line comparisons

Consortial variant (CVF) files of SNP and indel variation from four other zebrafish lines (AB, TU, TL, WIK), compiled through integration of data from three previous studies (Obholzer et al. 2012; Bowen et al. 2012; Butler et al. 2015), were downloaded from https://snpfisher.nichd.nih.gov/snpfisher/tracks.html. Each of these studies sequenced a pool of zebrafish between 3× and 16× coverage and aligned reads as one sample to the Zv9 reference genome for each line.

To compare T5D variant sites, the positions based on the GRCz10 reference genome needed to be mapped back to equivalent locations in the Zv9 build using Picard’s LiftoverVcf with the danRer10ToDanRer7 chain file from hgdownload.cse.ucsc.edu/goldenPath/danRer10/liftOver/. 20,131,988 SNPs and 5,630,544 indels were successfully mapped back to the Zv9 reference.

Additionally, the CVF files had masked variants in non-complex regions of the genome. To filter T5D variants accordingly, the repeat masked annotation of Zv9 was downloaded from http://hgdownload.soe.ucsc.edu/goldenPath/danRer7/database/rmsk.txt.gz. Approximately 51% of the genome is masked for having highly repetitive content. As a reference, over 56% of the human genome is masked (http://www.repeatmasker.org/). Variants located in these non-complex regions of the genome were removed from the final T5D comparison dataset resulting in 10,301,547 SNPs and 2,375,455 indels. To ensure consistency between datasets, we performed the same masking procedure on the AB, TU, TL, and WIK datasets even though masking had been previously performed. All comparisons with these lines were based on the following approximate counts (T5D: 10.3 M SNPs, 2.4 M indels; AB: 4.3 M SNPs, 0.6 M indels; TU: 3.6 M SNPs, 0.4 M indels; TL: 6.2 M SNPs, 0.8 M indels; WIK: 8.5 M SNPs, 1.1 M indels).

A VCF file for NHGRI-1 (LaFave et al. 2014) was downloaded for use in a separate line comparison due to sequencing strategy differences and alignment to different versions of the reference genome. The file included 17,089,212 variant calls (15,680,057 SNPs) and genotypes for the two founders based on high coverage individual whole genome sequencing and alignment to GRCz10 without masking. The VCF of 20,385,817 SNPs for T5D compared to the GRCz10 reference was used for SNP site comparisons to the NHGRI-1 line only.

### Downsampling

To address the impact of sequence design on comparisons between T5D and other lines that used pooled sequencing, a portion of the T5D data was used as a simulated pool. This was performed with the intention to more closely approximate variants that would have been called in T5D, had a pooled approach been employed instead of individual sequencing. First, 20 individuals were randomly selected. Next, 20% of each of their reads were randomly selected to create a simulated pooled sample at an average of 20× coverage. Alignment, variant calling, and filtering were all performed with the previous parameters. Before filtering, 18,086,779 SNPs were called. After filtering, 12,179,880 SNPs remained, of which 12,009,411 were successfully mapped to the Zv9 reference genome. For indels, the count decreased from 2,966,260 to 2,608,746 to 2,339,775. After masking variants located in non-complex regions of the genome, the final pooled approximation T5D comparison dataset resulted in 6,175,287 SNPs and 1,080,749 indels.

### T5D-specific reference

A T5D-specific reference was created. SNP and indel VCF files based on the GATK best practices recommendations were used. The indel file was further filtered to remove known repeats in the GRCz10 reference build. This minimized differences called based on microsatellites and other variable number tandem repeats (VNTRs). A bed file of all known *D. rerio* repeats was downloaded from the UCSC Genome Browser, containing 3,475,284 repeats of various types. These were screened out of the indel files to minimize the inclusion of microsatellite differences and other potential variants that may be more individual-based than population-based. The resulting VCF files were merged and used, in conjunction with the GRCz10 genome, as input for the GATK FastaAlternateReferenceMaker tool. A reference for the T5D population is available through GenBank (https://www.ncbi.nlm.nih.gov/genbank/).

The original and new genomes were split by chromosome for comparison using the nucmer package from the software MUMmer. Nucmer was run with the—mum option. The resulting delta files were filtered for 1-to-1 alignments allowing for rearrangements, and the filtered delta files translated to coordinates to be used in MapView for plotting. The filtered delta files were also run through dnadiff.

## Results

### Interspecies comparisons

The zebrafish genome (1.5 Gb) is roughly half the size of the human (3.3 Gb) or mouse (2.8 Gb) genome. To date, the total number of discovered variants in the zebrafish genome is less than half the number found in human or mouse genomes; consequently, validation is more sparse. The allele frequency distribution of “common” human variants indicates that the majority of common variants are infrequent across the overall human population [minor allele frequency (MAF) < 0.1] (Fig. 1b). Though these SNPs are private to all save a handful of people, they are only prevalent in specific subpopulations. The majority of common variants in the human genome have already been discovered, but rare variants continue to be discovered via deep whole genome sequencing of cohorts of individuals from geographically/ethnically defined populations (Shen et al. 2013).

### T5D variants

The estimate of 20.1 M SNPs segregating in the population (10.3 M in non-repetitive regions of the genome used for zebrafish line comparisons) included non-reference allele frequencies from 0.1 to 99.8%. We posit that the 10.3–20.1 M SNPs and 2.8–5.6 M indels discovered in T5D are accurate bounds for an estimate of variability in this zebrafish line. With more individuals and higher coverage, we would expect to find even more rare variants segregating in the population. This would be consistent with the continued rare variant discovery in human populations noted in the previous section.

With the exception of chromosome 4, the number of variants discovered per chromosome was proportional to chromosome length (Appendix Table 1). There was a region of chromosome 4 with drastically fewer variants in our study (Appendix Fig. 4) that was also reported in (Butler et al. 2015). This low-variability region lies within an area of the genome that has primarily zebrafish-specific genes not homologous to other species (Howe et al. 2013). There is evidence that chromosome 4 is involved in sex determination in natural zebrafish populations (Wilson et al. 2014).

### Interspecies comparisons to T5D zebrafish

The proportion of the types of SNP found in T5D were similar to those reported by the dbSNP variant sites in both human and mouse. We observed more intron variants in T5D, and synonymous gene transcript variant percentages fell between mouse and human (Fig. 1a). The larger percentage of intronic variants in zebrafish can be explained by genetic architecture, as the value is proportional to the percent of the genome sequence that is intronic (roughly 43.9% of the zebrafish genome, 39.6% of the human genome, and 26.6% of the mouse genome) (Sakharkar et al. 2005; Moss et al. 2011).

The 20.1 M SNPs equate to 13.4 SNPs per 1 kb genomic sequence. Prior studies estimated that certain zebrafish strains contained an average of 7 SNPs per 1 kb of non-repetitive (i.e., non-complex, non-masked) genome sequence per strain, which is still more than in any ethnically defined human population from the 1000 Genomes (Butler et al. 2015). Estimates in other species have been similar (4.9 SNPs per kb in sheep, 5.5 SNPs per kb in chickens, 10.1 SNPs per kb in fly, and 13.9 SNPs per kb in mouse), though they have been based on combined line/breed data (Ka-Shu Wong et al. 2004; Kijas et al. 2009; Kang et al. 2016; Srivastava et al. 2017).

On average, an individual in the T5D population was found to carry a non-reference allele (homozygous non-reference or heterozygous) at 6.9 M SNP sites and 1.8 M indel sites (3.7 M SNP sites and 0.84 M indel sites in non-masked genomic regions). This is more than have been identified in individual human genomes. For example, in Caucasians an average of 3.3 M SNPs and 0.49 M indels with non-reference alleles were identified per individual (Shen et al. 2013). In Turkish individuals, an average of 3.3 M SNPs and 0.91 M indels were identified (Alkan et al. 2014). In Chinese individuals, an average of 3.5 M SNPs and 0.63 M indels were identified (Shi et al. 2016). Comparing across broad populations, Cho et al. found an average of 4.6 M SNPs and 0.68 M indels per African individual, 3.75 M SNPs and 0.60 M indels per Caucasian, and 3.69 M SNPs and 0.54 M indels per Asian. When using a Korean genome as the reference, the number of calls increased for each of the African and Caucasian individuals and decreased for the Asian individuals (Cho et al. 2016).

The abundance of sites with non-reference alleles per T5D zebrafish could imply that within a population, zebrafish are more genetically variable than humans. However, because ethnic/population-level choice of reference may influence the number of variants called (Cho et al. 2016), an individual zebrafish within the T5D population may vary more from the current zebrafish reference genome than individuals from certain human ethnic populations vary compared to the human reference genome. While this could indicate that the human reference genome provides a more representative consensus across human populations, it is also possible that the absence of admixing between zebrafish laboratory populations may have caused them to diverge more from a historical reference sequence.

### Zebrafish line comparisons

**Fig. 2:**
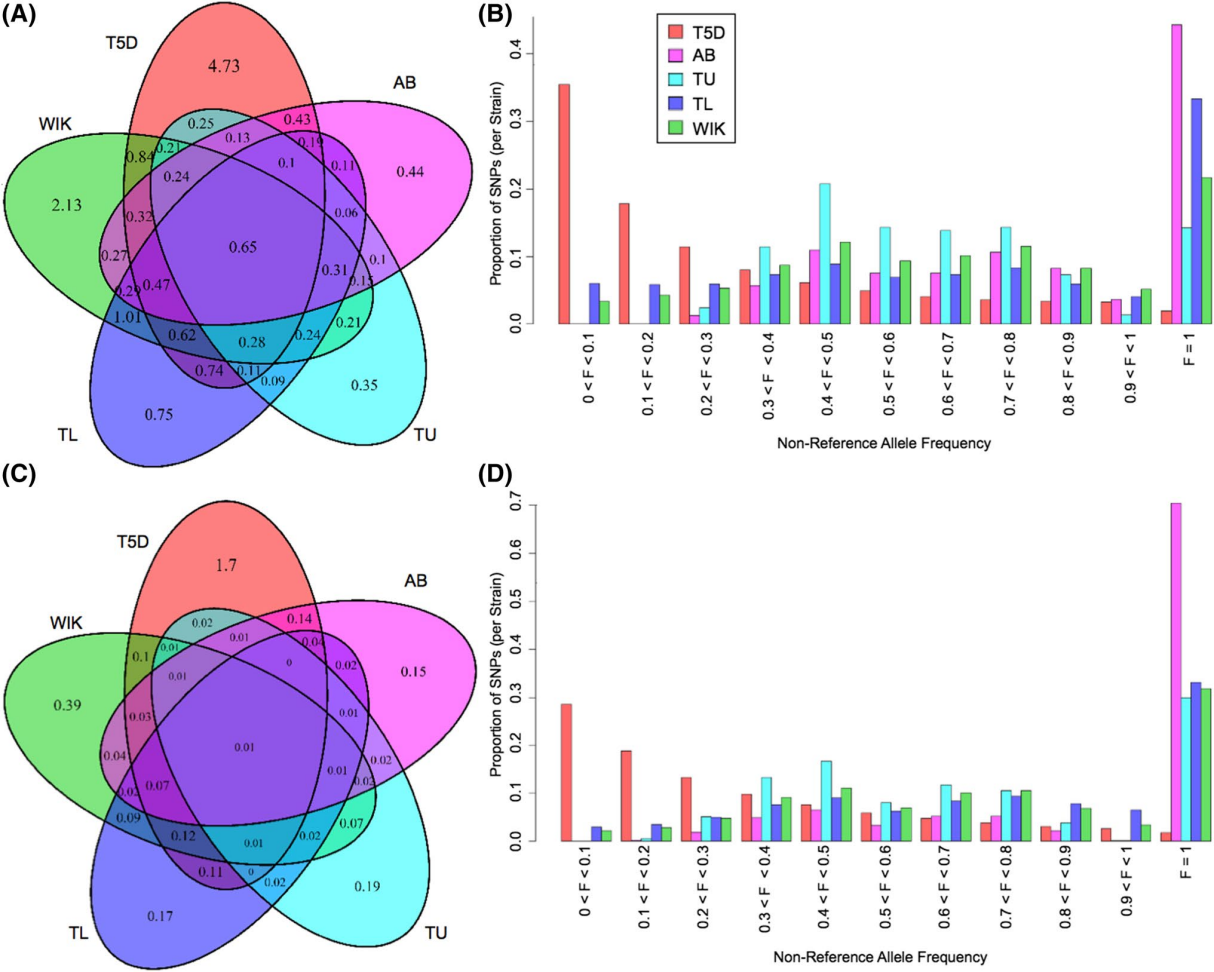
Zebrafish variant comparisons. **a** Venn diagram of SNP sites (in millions) compared to the Zv9 reference genome. **b** Proportions of SNPs binned by alternate allele frequencies for the 5 lines. The T5D allele frequencies are based on 276 individual whole genome sequences. For all other lines, frequencies were determined based on the proportion of reads with non-reference base calls since no individual genotypes can be determined from pooled sequence alignment. **c** Venn diagram of indel sites (in millions). **d** Proportion of indels for discrete alternate allele frequencies

T5D was found to have more variants compared to results from studies using pooled sequencing and smaller sample sizes (Fig. 2a, c). T5D variants, discovered based on approximately 1380× coverage across individuals (5× for 276 individuals), followed an allele frequency spectrum more similar to known human variants (Figs. 1b, 2b, d). Variants discovered in the other lines in pooled sequencing experiments were primarily common, because a given site had low coverage (< 20×) across the pool. Additionally, rare variants (those observed at frequencies of < 0.1) would have been missed at small sample sizes. For T5D, the plurality of the variants discovered were rare.

The comparator lines displayed an abundance of fixed mutations versus the reference genome that were not observed in T5D. This can also be explained by small sample size and coverage in a pooled sample. Many of these sites may actually be variable in the populations (rather than fixed) yet missed in the sampled subsets.

For the previously discovered variants in AB, TU, TL, and WIK, SNPs in TU followed a slightly different read frequency distribution, with fewer fixed SNPs. This can be explained in part by the heavy reliance of the reference genome sequence on TU zebrafish. Additionally, AB and TU had even fewer low-frequency SNPs, which can be explained by the lower average read depth per SNP site (median of 8 for AB and 9 for TU compared with 16 for TL and 13 for WIK).

In order to assess the similarity of T5D variation to a hybrid population that has previously employed an individual sequencing approach, SNP sites were compared to NHGRI-1 SNP sites. The NHGRI-1 line was derived from one mating pair of TAB-5 (a TU and AB cross), where the founding male was previously sequenced at 52× coverage and the female at 47× (LaFave et al. 2014). Even with the small sample size of 2, 15.7 M SNPs were discovered, with more than 10 M novel (i.e., not in dbSNP). Of these, 6.85 M overlap with the SNPs discovered in T5D.

Though more SNPs were found in a T5D sample that included more individuals, the two NHGRI-1 founders carried non-reference alleles at more sites (an average of 12.8 M variant sites per individual compared to 6.9 M in T5D). This may be partially explained by the lower coverage per individual in our design, wherein we sacrificed sequencing depth per individual in order to include a larger sample and better estimate genotype frequencies for rare variants. These rare variants would not be captured without a reasonably large sample of individuals.

### Downsampling to approximate sequencing designs in other lines

In order to assess whether sequencing design could be a major driver behind observed SNP differences between lines, we used a downsampling strategy to approximate published designs used for other lines. We simulated a pool of 20 T5D individuals with average coverage of 20× across the genome by using a subset of the sequencing reads and analyzing them as one pooled sample. Even before applying filters, 49.8% as many variants were detected in this pooled sample compared to the whole dataset. After the simulated analysis, median read depth per variant site for T5D was 14 (within the range of 8–16 mentioned previously for the other 4 lines).

**Fig. 3:**
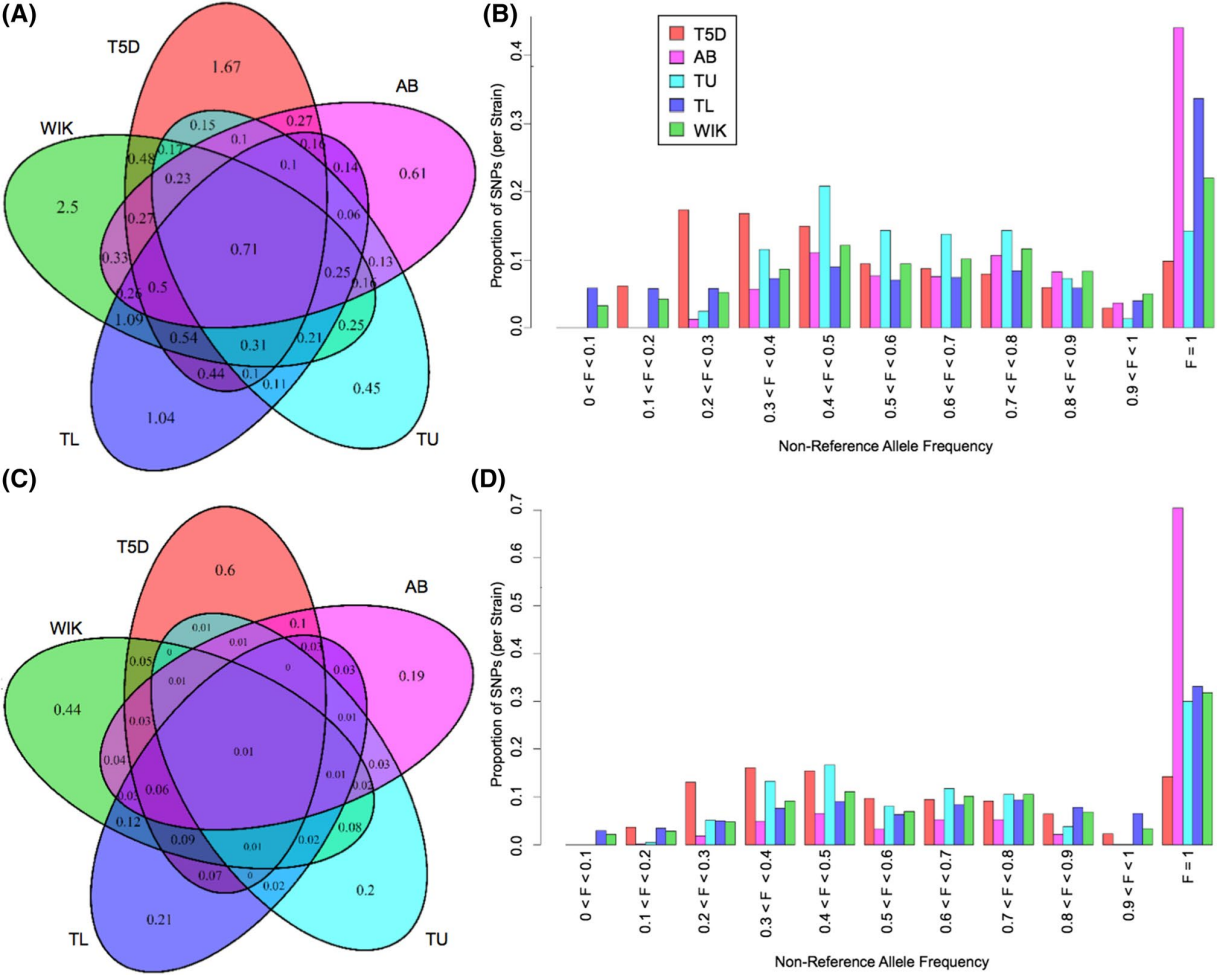
Zebrafish variant comparisons after sequencing and masking a pooled subsample. **a** Venn diagram of SNP sites (in millions) compared to the Zv9 reference genome. **b** Proportions of SNPs binned by alternate allele frequencies for the 5 lines. For all lines, frequencies were determined based on the proportion of reads with non-reference base calls since no individual genotypes can be determined from pooled sequence alignment. **c** Venn diagram of indel sites (in millions). **d** Proportion of indels for discrete alternate allele frequencies

T5D variant counts and proportions of non-reference reads moved closer to those observed in other lines (Fig. 3). Low-frequency variants were no longer identifiable, and a larger proportion of the non-reference alleles incorrectly displayed themselves as fixed mutations (F = 1 in Fig. 3b, d). This downsampling approach resulted in a twofold reduction in variant calling capability, providing evidence that sequencing design could be a major driver of variability differences among zebrafish lines.

## Discussion

We used new data from a genome-wide sequencing project to compare and characterize observed population genetic variation across species (humans, mice, zebrafish). While more variants have been discovered in the human and mouse genomes, the smaller zebrafish genome is on par with—or in some cases may even exceed—genetic variability observed between individuals in those species. This diversity is attractive for translational applications in human and ecological health, where natural genetic variability could manifest as susceptibility differences to chemicals, drugs, environmental change, or other stressors. Though there are fewer zebrafish disease models compared to other species (Fig. 1c), the number of genetic associations for many phenotypes of interest in health and environmental studies in zebrafish follows sequentially after human and mouse (Fig. 1d). Indeed, the zebrafish model is gaining tractability as a human disease model (Howe et al. 2017).

Variant discovery in the T5D wild-type zebrafish has confirmed the line’s status as a heterogenous population. Considerably more SNPs and indels were discovered through individual whole genome sequencing of a large T5D sample than in other zebrafish studies, even exceeding the current build of dbSNP. Pooled sequencing data fundamentally affected the character of genetic variation previously detectable in outbred zebrafish lines, versus the individual-level sequencing data collected for T5D. In addition to discovering more variants, the design allowed us to estimate allele frequencies for a population more accurately than previously possible due to bias when estimating based on read frequencies in a pool (Raineri et al. 2012) or sample size of 2. Subsampling to simulate a pooled sequencing approach showed that T5D variation is in line with the more variable zebrafish laboratory strains (Fig. 3). This likely means that (1) many of the variants discovered in T5D are present in other lines as well but have not been found due to pooling, low coverage, and sample size restrictions in previous zebrafish experiments, and (2) there are many more rare alleles that are yet to be discovered. This latter trend is very similar to continued improvements in rare allele discovery in humans (Shen et al. 2013). Our observations suggest that interindividual genetic diversity (i.e., natural variation) within laboratory populations may be higher than currently estimated and may have implications for differential susceptibility observed in toxicological studies.

For environmental health research, this means that healthy laboratory zebrafish strains that are sufficiently outbred, and thus of comparable genetic diversity versus other natural populations, can be a powerful model for environmental exposure studies in humans and other species. Their rapid development allows for high-throughput studies that can expand scientific discovery on several axes related to differential susceptibility. Because select individuals or entire communities may be especially susceptible to adverse health effects from chemical exposure through common consumer products, occupational hazards, environmental emergencies, or geographic location (Brette et al. 2014; Judson et al. 2010), models for diverse populations are needed to explore this interindividual susceptibility (French et al. 2015). Continued work on identifying genetic variation in commonly used zebrafish lines will be important for exploration of gene–environment interactions (G×E), epigenetic modifications, and other genetic effects linked to environmental exposure-associated hazards.

There are also long-term benefits associated with creating a database of known SNPs in zebrafish populations. This database of population genomic information can inform future research and can be expanded in later phases and through other projects. Changes in genotype frequencies within the population can be tracked, which can address whether genetic drift or unwanted selection is affecting a laboratory population aiming to maintain an “outbred” strategy that maintains diversity.

Additionally, population genetic information can be used to determine variants (SNPs, copy-number variants, etc.) associated with differential chemical responses (Balik-Meisner et al., *submitted*). Risk assessment can be improved significantly with actual knowledge of subgroup and chemical-specific genetic variability (e.g., confidence bounds or upper/lower limits) (Dankovic et al. 2015; Schulte et al. 2015; Betts and Shelton-Davenport 2016). This is true for applications that range from environmental chemical exposure studies or pharmaceutical trials in human populations to environmental emergencies affecting ecological species, such as the response to the spill of MCHM in West Virginia (http://ntp.niehs.nih.gov/results/areas/wvspill/studies/index.html). Thus, inclusion of knowledge regarding constitutive genetic diversity will benefit all translational applications of the zebrafish model, from the mechanistic to the ecological to the clinical.

## Figures and Tables

**Fig. 4:**
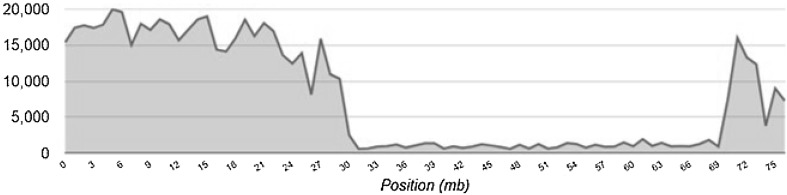
Distribution of variants on chromosome 4. The* y* axis displays the variant count partitioned into 1 mb bins of genomic sequence (*x* axis)

## Appendix

See Fig. 4 and Table 1.

**Table 1:** SNP count per chromosome

Chromosome	SNP count	Chromosome length (bp)	SNP percentage
1	937,216	58,871,917	1.59
2	992,016	59,543,403	1.67
3	903,306	62,385,949	1.45
4	593,111	76,625,712	0.77
5	1,123,780	71,715,914	1.57
6	1,010,933	60,272,633	1.68
7	1,071,615	74,082,188	1.45
8	796,793	54,191,831	1.47
9	928,007	56,892,771	1.63
10	695,209	45,574,255	1.53
11	664,127	45,107,271	1.47
12	708,967	49,229,541	1.44
13	789,917	51,780,250	1.53
14	894,307	51,944,548	1.72
15	756,502	47,771,147	1.58
16	861,924	55,381,981	1.56
17	860,076	53,345,113	1.61
18	817,743	51,008,593	1.60
19	783,706	48,790,377	1.61
20	854,683	55,370,968	1.54
21	726,643	45,895,719	1.58
22	596,605	39,226,288	1.52
23	763,643	46,272,358	1.65
24	677,988	42,251,103	1.60
25	577,000	36,898,761	1.56
